# Simultaneous Measurements of Nanotrace Amounts of Lead and Cadmium Using an Environmentally Friendly Sensor (An Activated Glassy Carbon Electrode Modified with a Bismuth Film)

**DOI:** 10.3390/molecules30061308

**Published:** 2025-03-14

**Authors:** Katarzyna Tyszczuk-Rotko, Aleksy Keller

**Affiliations:** Faculty of Chemistry, Institute of Chemical Sciences, Maria Curie-Skłodowska University in Lublin, 20-031 Lublin, Poland; alekskeller@op.pl

**Keywords:** lead and cadmium measurements, environmentally friendly sensor, activated glassy carbon electrode with in situ deposited bismuth film, stripping voltammetry, water samples

## Abstract

This paper shows the fabrication of a new environmentally friendly sensor, an activated glassy carbon electrode with an in situ deposited bismuth film (aGCE/BiF), to determine Cd(II) and Pb(II) at the nanotrace level. The electrochemical activation of the GCE surface was achieved in a solution of 0.1 M phosphate-buffered saline (PBS) of pH = 7 by performing five cyclic voltammetric scans in the range of −1.5–2.5 V at ν of 100 mV/s. The newly developed electrode provides several advantages, such as an increased electron active surface (compared to the glassy carbon electrode) and improved electron transfer kinetics. As a result, the new voltammetric procedure (square-wave anodic stripping voltammetry, SWASV) was established and optimized. With the SWASV method, the following calibration curves and low detection limits (LODs) were obtained for Cd(II) and Pb(II), respectively: 5–100 nM, 0.62 nM, 2–200 nM, and 0.18 nM. The newly prepared method was used to determine the amounts of Pb(II) and Cd(II) in the certified reference material, and the results agreed with the certified values. Moreover, the procedure was successfully applied to determine the Cd(II) and Pb(II) in river samples. The official and standard addition methods validated the measurement results.

## 1. Introduction

Heavy metals are a group of substances that pollute the environment. They are characterized by a high molar mass, high density, and toxicity to living organisms. Typical examples are copper, lead, nickel, iron, and cadmium. They are highly toxic and remain in the atmosphere for a long time, so they can accumulate in the human body through so-called bioaccumulation. These compounds may enter terrestrial and aquatic environments, causing pollution, and once they enter some environments, they are very difficult to completely remove. The aquatic environment can be polluted with heavy metals in many ways, including the burning of fossil fuels, vehicle exhaust gases, mining, agriculture, and the burning of solid and liquid waste. In the air, heavy metals can occur naturally due to the activities of volcanoes, thermal springs, erosion, infiltration, etc. Over the last few years, there has been a lot of interest in studying the impacts of these toxic agents because human health is ubiquitously exposed to the effects of these compounds. Unfortunately, heavy metals are commonly used in everyday life, for example, in electrical devices and industry, and environmental pollution with these metals has become a serious problem. In general, the toxicity of metal ions is due to their chemical nature. They can react with cellular structural proteins, enzymes, and membrane systems and can block their biological activity. They can enter the organs of a living organism and accumulate, and the amount of metal that can be deposited depends on the route of exposure and the chemical properties of the metal such as its valency state, volatility, lipid solubility, etc. Recently, it has been shown that some heavy metals such as chromium, nickel, or lead may contribute to the development of cancer. In this paper, we would like to focus primarily on the negative impact of cadmium and lead [1,2,3].

People are exposed to cadmium through inhalation of polluted air and ingestion. The kidneys and bones are mainly at risk, where cadmium can accumulate. This metal can also cause lung damage, mainly in tobacco smokers. For non-smokers, cadmium poisoning may occur due to an improper diet (90% of cases for non-smokers). However, in the aquatic environment, cadmium is not degraded to less toxic products, which contributes to its bioaccumulation in the kidneys and livers of aquatic organisms and microflora. Interestingly, it is believed that cadmium is not necessary for the proper functioning of the human organism [2]. It is worth mentioning that cadmium can lead to an imbalance of some minerals in the human body, such as zinc, magnesium, or copper [4]. Lead, however, can cause lung cancer or stomach cancer through repeated exposure, which causes the concentration of lead in the blood to increase rapidly. Studies have also examined possible links between workplace exposure to lead and other cancers such as brain, kidney, bladder cancer, etc., but the results were mixed. Additionally, lead can cause anemia and problems with the nervous system [2]. Moreover, the FAO/WHO talks about the PTWI index, which stands for Provisional Tolerable Weekly Intake. This is the amount of a substance that does not have harmful effects on the human body. For cadmium and lead, the PTWI values are, respectively, 7 μg/kg and 25 μg/kg per kilogram bodyweight [5]. Pollution with heavy metal ions and their negative impact on living organisms are a serious environmental problem, which is why it is so important to control the concentration of these ions, in particular cadmium and lead, at the trace level.

Several analytical methods for simultaneously determining cadmium and lead are described in the literature, mainly spectroscopic ones, for example, graphite furnace atomic absorption spectrometry [6], inductively coupled plasma mass spectrometry [7], or flame atomic absorption spectrophotometry [8], in different matrices. However, voltammetric methods predominate in the vast majority of cases. Compared to spectroscopic methods, voltammetric measurements do not use expensive and complicated equipment [9]. Moreover, voltammetric methods are characterized by much higher sensitivity, faster measurements, and lower limits of detection (LODs) and quantification (LOQs). It is also worth mentioning that by using special chemical sensors, e.g., screen-printed electrodes (SPEs), you can further miniaturize the equipment and perform field analyses. Recently, there has been a tendency to use sensors whose surface has previously been modified [10,11,12,13,14,15]. This can be achieved by electrochemical deposition of a metal film, e.g., bismuth film (BiF) [11], or surfactant film such as sodium dodecylsulphate (SDS) [16], deposition of nanoparticles such as silver nanoparticles (AgNPs) [15], or polymerization of a given chemical compound [11]. Furthermore, an equally common step during sensor pre-preparation is electrochemical activation. There are many types of activation, such as treatment with a laser, vacuum heating, mechanical polishing, ultrasonication, or the carbon arc, and most of them are very-well known. Recent studies have shown that sensors such as glassy carbon electrodes (GCEs) can easily be activated by pre-anodization, which is followed by short cathodization, cathodization, or anodization at high potential. Such preparation can create surface functional groups (SFGs) [17,18]. All these steps are intended to improve the parameters that describe the electrode, such as the electron active surface, kinetics of electron transfer, or charge transfer resistance (R_ct_). Moreover, activation can increase the polarity of the electrode surface.

There has been great interest in bismuth-based sensors [12,19,20,21,22,23]. These electrodes have partially replaced mercury electrodes such as hanging mercury drop electrode (HMDE) or mercury film electrodes (MFEs) due to their toxicity and volatility. Sensors modified with bismuth-based materials feature properties such as a low background current, large active surface, and low charge transfer resistance. Most importantly, these electrodes are non-toxic for humans and do not cause lasting effects. In addition, bismuth-based modifiers can increase the selectivity of a voltammetric method for simultaneous determination of cadmium and lead ions due to better peak separation, e.g., reducing thallium interference [23]. Data found in the literature indicate that bismuth modifiers significantly improve the sensitivity of cadmium ion determination, and increasing the polarity of the carbon surface improves the sensitivity of lead ion determination. This is because the more favored interactions are Cd-bismuth and Pb-polar electrode surface, which can be seen in the case of determining the traces of these ions without the use of modifiers (no signal from Cd(II) and a small signal from Pb(II) on unmodified carbon electrodes) [19].

Previous methods described in the literature give results comparable to those obtained in this work (Table 1) [9,10,11,12,13,14,15,19,20,21,22,23,24,25,26,27,28]. This raises the question of where the novelty of this paper lies. All previous methods described in the literature are characterized by complicated steps in preparing the chemical sensor, long times of accumulation of the analyte on the electrode surface, or the usage of chemical reagents that are dangerous/toxic to humans or pollute the environment and cause lasting effects. To overcome those issues, we propose a method in which sensor activation is quick and simple and the measurements are characterized by simplicity, speed, and the use of non-toxic reagents such as bismuth nitrate or phosphate-buffered saline. To study the electrochemical properties of the aGCE/BiF, CV scans using the Fe(III)/Fe(II) redox system were performed. In addition, scanning electron microscopic (SEM) measurements were taken to identify differences in changes on the electrode surface before and after activation.

## 2. Results and Discussion

### 2.1. Activation of the Electrode Surface

**Figure 1:**
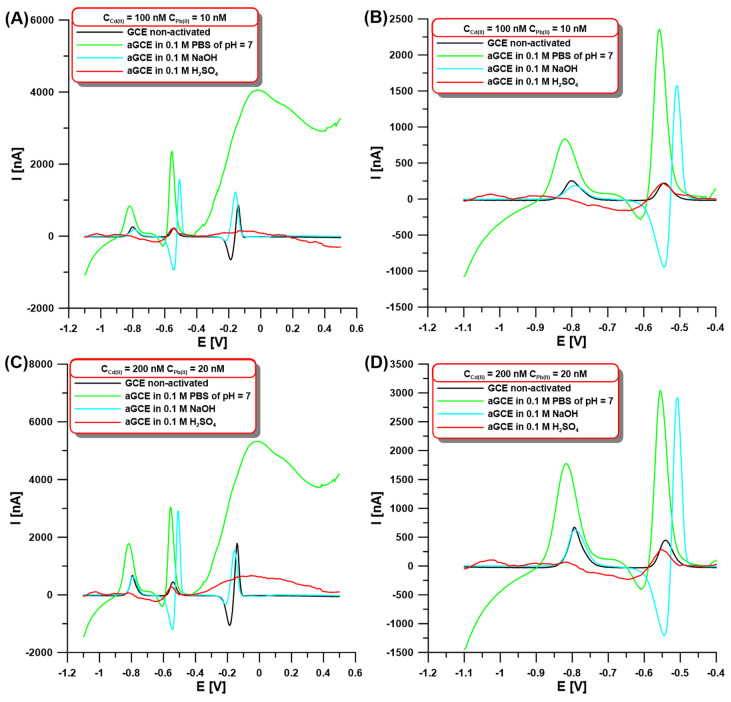
The SWASV at the GCE/BiF and aGCE/BiF activated in 0.1 M PBS of pH = 7 (green curves), 0.1 M NaOH (blue curves), and 0.1 M H_2_SO_4_ (red curves) in the presence of 100 nM Cd(II) and 10 Pb(II) (**A**,**B**) or 200 nM Cd(II) and 20 nM Pb(II) (**C**,**D**). The activation was performed using 5 CVs in the potential range of −1.5–2.5 V at ν of 100 mV/s The SWASV conditions: 0.1 M acetate buffer of pH = 4.5, 3.0 µM Bi(III), electrochemical cleaning of the electrode surface at 0.5 V for 60 s, E_dep.Bi+acc._ of −1.1 V, t_dep.Bi+acc._ of 60 s, f of 25 Hz, E_SW_ of 25 mV, and ΔE of 4 mV. The SWASV curves were registered in the potential range of −1.1–0.5 V (from (**A**) to (**D**)). (**B**,**D**) are cutouts (−1.1–(−0.4 V)) of (**A**) and (**C**), respectively.

In the initial stage of the experiments, the SWASV signals of Cd(II) and Pb(II) were compared using the GCE modified with a bismuth film at non-activated (GCE/BiF) and activated electrodes on different solution surfaces (aGCE/BiF). Our goal was to check whether electrochemical activation in other solutions (0.1 M PBS of pH = 7, 0.1 M NaOH, and 0.1 M H_2_SO_4_) contributes to changes in the peak current intensity of the determined ions. The activation was performed using five cyclic voltammetric (CV) scans in the potential range of −1.5–2.5 V at a scan rate (ν) of 100 mV/s. The choice of solutions and the activation procedure were not accidental since they were based on literature data [18,29]. SWASV voltammograms were registered on both the GCE/BiF and aGCE/BiF electrodes in 0.1 M acetate buffer of pH = 4.5 containing 3.0 µM Bi(III) and 100 or 200 nM Cd(II), and 10 or 20 nM Pb(II) after 60 s accumulation at the potential of −1.1 V (Figure 1). The obtained results for both the lower and higher concentrations of Cd(II) and Pb(II) clearly show that the highest analyte signal can be obtained on the sensor activated in 0.1 M PBS of pH = 7 (Figure 1A–D, green vs. black curve). The SWASV curves (Figure 1) show peaks of Cd (around −0.8 V), Pb (around −0.55 V), and Bi (around 0 V). Small changes in the peak potentials depending on the composition of the solution used for the activation electrode were observed. Additionally, in the case of sulfuric acid, an additional peak at around −1.05 V is visible, which is related to the contamination of the electrode surface during the activation stage. The activation causes an increase in the peak current intensity of cadmium up to 283.6% for 100 nM Cd(II) and 255.6% for 200 nM Cd(II) compared to the signals obtained on the non-activated GCE/BiF. On the other hand, in the case of the lead signal, increases of up to 1233.2% (for 10 nM Pb(II)) and 856.1% (for 20 nM Pb(II)) were observed after electrochemical activation. The increase in the signals can be explained by the improvement of the electron transfer ability and enhancement of the electrochemical activity of the sensor, which is connected with the formation of oxygen-containing surface functional groups. The formation of these groups after electrochemical activation in 0.1 M PBS of pH = 7 on the GCE surface has been well-described in the literature [17,18].

**Figure 2:**
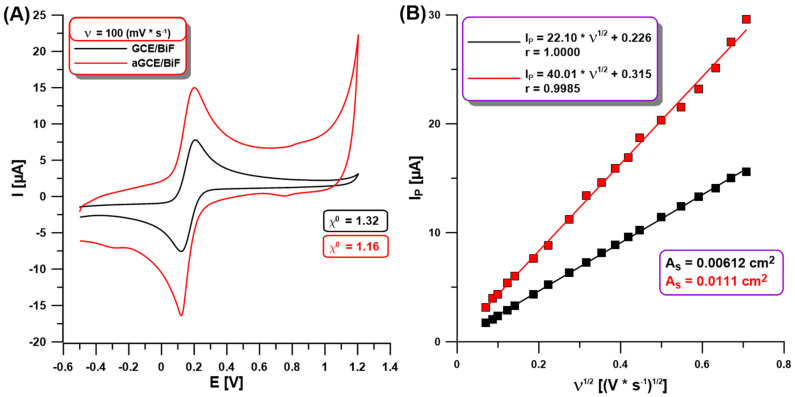
(**A**) CVs at the GCE/BiF (black curve) and aGCE/BiF (red curve) using ν of 100 mV/s in 0.1 M KCl containing 0.5 mM [Fe(CN)_6_]^3−/4−^; (**B**) dependence between the anodic I_p_ and the square root of ν (ν^1/2^, ν of 5–500 mV/s) at the GCE/BiF and aGCE/BiF activated in 0.1 M PBS solution of pH = 7.0 ± 0.1 by 5 CVs in the potential range of −1.5–2.5 V (ν of 100 mV/s).

In our studies using CV in a solution of 0.1 M KCl containing 0.5 mM [Fe(CN)_6_]^3−/4−^ at the GCE/BiF and aGCE/BiF activated in 0.1 M PBS of pH = 7, we showed changes between the electrochemical properties of both sensors. Figure 2A demonstrates the CVs on the non-activated GCE/BiF and activated aGCE/BiF at ν of 100 mV/s. The enhancement of the analytical signal of Fe(II) and Fe(III) at the activated electrode is visible in comparison to the non-activated one (Figure 2A,B). The calculated relative peak separation (χ^0^) values (1.32 for GCE/BiF vs. 1.16 for aGCE/BiF) and the electrochemically active areas (As) of the electrodes (0.00612 cm^2^ for the GCE/BiF vs. 0.0111 cm^2^ for the aGCE/BiF) clearly show an improvement in the electrochemical properties (improvement in the electron transfer between the electrode surface and the solution, and increasing the number of active sites where electrode reactions can occur) after electrochemical activation in 0.1 M PBS of pH = 7.

**Figure 3:**
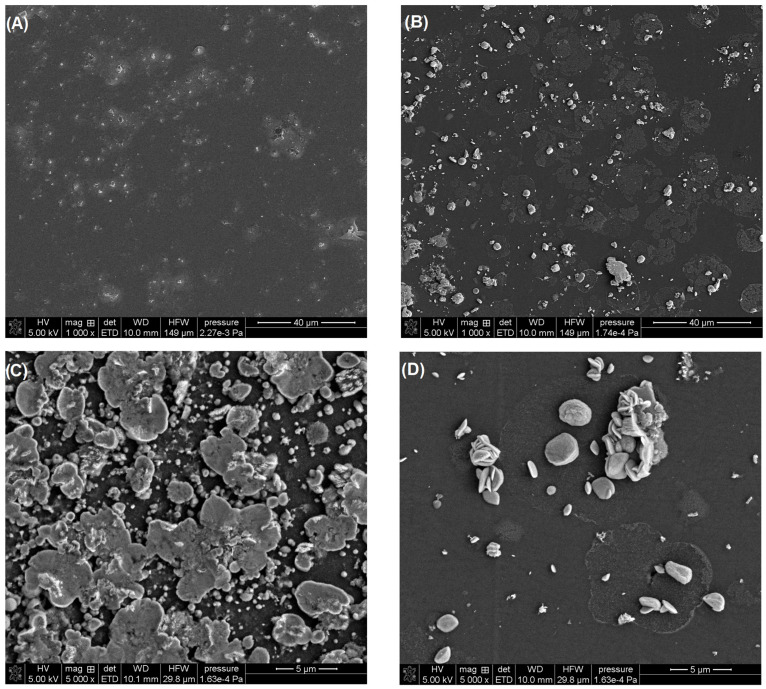
SEM images of the GCE/BiF (**A**) and aGCE/BiF (**B**–**D**).

In our studies, we confirmed that the activation of the electrode in 0.1 M PBS of pH = 7 contributes to a change in the morphology of the deposited bismuth film. In the SEM images (Figure 3), significant differences can be seen in the structure of the BiF deposited onto the non-activated and activated electrode surfaces as well as in the number of particles on the surfaces (Figure 3A vs. Figure 3B). On the aGCE/BiF, bismuth particles take on various shapes, from mushroom-like formations to overlapping plates (Figure 3C,D), and the film structure is more developed since we observe a much greater number of these particles on the surface.

**Table 1:** Comparison voltammetric procedures for the Cd(II) and Pb(II) quantification.

Electrode	Technique	Linear Range [nM]		LOD [nM]		Analyzed Sample	Ref.
		Cd(II)	Pb(II)	Cd(II)	Pb(II)		
HMDE	DPAdCSV	1.8–266.9	2.4–337.8	0.089	0.082	Food	[9]
CMCPE	DPASV	250.0–25,000.0	100.0–15,000.0	40.0	10.0	Water	[10]
SPCE/poly(BCP)/Bi	DPASV	0–2224.0	0–1207.0	0.32	0.13	Wastewater	[11]
GCE/BiF/NanoSiO_2_	SWASV	17.8–1334.0	9.7–724.0	5.34	0.96	Water	[12]
BDDE	SWASV	50.0–3985.0	48.0–7954.0	30.0	17.50	-	[13]
GCE/MoSI	DPASV	4.5–1334.0	7.2–724.0	0.89	2.17	Tap water	[14]
GNs@AgNP	SWASV	21.3–192.0	198.0–989.0	0.21	0.21	Water	[15]
GCE/NHgFE	SWAdSV	4.5–356.0	2.4–193.0	1.78	0.96	CRM	[25]
GCE/HgF-N-L	SWASV	16.0–222.0	1.9–145.0	0.089	0.39	Tap water	[26]
GE/poly(PCA)	SWASV	356.0–8896.0	193.0–4826.0	137.0	66.0	Freshwater and water	[27]
GCE/MnCo_2_O_4_NPs	LSASV	50.0–1600.0	50.0–40,000.0	7.02	8.06	Distilled, tap, and river water	[28]
GSPE/Bi_2_O_3_	SWASV	17.8–177.9	9.6–96.5	15.10	2.41	Groundwater	[19]
GSPE/CS@Bi_2_O_3_	SWASV	17.8–177.9	9.6–96.5	13.30	0.96	Groundwater	[19]
SPGE/Nafion/BiF	SWASV	444.8–2668.8	96.5–1447.9	35.60	14.50	River water	[20]
GCE/Au-GN-SeCys/BiF	SWASV	4.5–889.6	2.4–482.6	0.71	0.24	Groundwater, soil, and plant samples	[21]
GCE/Bi_2_O_3_@NPBi	SWASV	4.5–1779.2	2.4–965.3	0.27	0.10	Tap water	[22]
CFME/BiF	SWASV	357.1–1785.7	193.1–965.3	-	5.31	-	[23]
GCE/BiF	DPASV	0–356.0	0–241.0	0.80	0.77	Water	[24]
aGCE/BiF	SWASV	5.0–100.0	2.0–200.0	0.62	0.18	CRM and river water	This work

HMDE—hanging mercury drop electrode; CMCPE—chemically modified carbon paste electrode (with diacetyldioxime); SPCE/poly(BCP)/Bi—bismuth/poly(bromocresol purple) modified screen-printed carbon electrode; GCE/BiF/NanoSiO_2_—glassy carbon electrode modified with bismuth film coated with mesoporous silica nanoparticles; BDDE—boron-doped diamond electrode; GCE/MoSI—glassy carbon electrode modified with Mo_6_S_9−x_I_x_ nanowires; GNs@AgNP—graphene decorated with silver nanoparticles; GCE/NHgFE—glassy carbon electrode modified with nafion-coated mercury film; GCE/HgF-N-L—glassy carbon electrode modified with mercury film coated with nafion-macrocyclic ester; GE/poly(PCA)—graphite electrode modified with poly(p-coumaric acid); GCE/MnCo_2_O_4_NPs—glassy carbon electrode modified with mnco_2_o_4_ nanoparticles; GSPE/Bi_2_O_3_—screen-printed graphite electrode modified with bismuth oxide; GSPE/CS@Bi_2_O_3_—screen-printed graphite electrode modified with chitosan and bismuth oxide composite; SPGE/Nafion/BiF—screen-printed golden electrode modified with nafion and bismuth film; GCE/Au-GN-SeCys/BiF—glassy carbon electrode modified with golden nanoparticles, graphene and selenocystheine composite, and bismuth film; GCE/Bi_2_O_3_@NPBi—glassy carbon electrode modified with bismuth oxide and nanoporous bismuth; CFME/BiF—carbo fiber electrode modified with bismuth film; GCE/BiF—glassy carbon electrode modified with bismuth film; DPAdCSV—differential-pulse adsorptive cathodic stripping voltammetry; DPASV—differential-pulse anodic stripping voltammetry; SWASV—square-wave anodic stripping voltammetry; SWAdSV—square-wave adsorptive stripping voltammetry; LSASV—linear sweep anodic stripping voltammetry.

### 2.2. pH and Concentration Studies of Supporting Electrolyte

**Figure 4:**
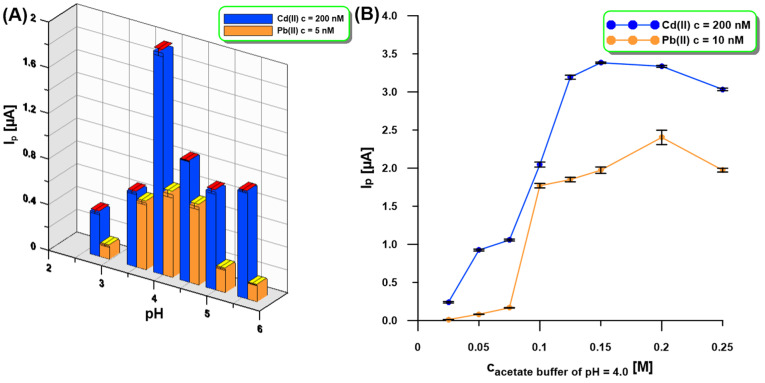
The influence of pH ((**A**), 0.1 M acetic acid, 0.1 M acetate buffer of pH = 3.5, 4.0, 4.5, 5.0, and 5.6) and concentration ((**B**), acetate buffer of pH = 4.0) of the supporting electrolyte on the SWASV response of Cd(II) (200 nM—(**A**,**B**)) and Pb(II) (5 nM—(**A**), 10 nM—(**B**)). Other SWASV conditions as in Figure 1.

The influence of the supporting electrolyte pH (0.1 M acetic acid, 0.1 M acetate buffer of pH = 3.5, 4.0, 4.5, 5.0, and 5.6) on the SWASV response of Cd(II) (200 nM) and Pb(II) (5 nM) at the aGCE/BiF activated in 0.1 M PBS of pH = 7 was tested (Figure 4A). It was found that pH has an impact on the peak current (I_p_) of Cd(II) and Pb(II) because the I_p_ values increased up to pH = 4.0 and then decreased. The next step was to study the influence of acetate buffer of pH 4.0 in the concentration range of 0.025–0.25 M (Figure 4B). The highest SWASV response of Cd(II) (200 nM) and Pb(II) (10 nM) was obtained in 0.2 M acetate buffer with pH = 4.0 and therefore it was recommended for further studies.

### 2.3. Bi(III) Concentration and Deposition/Accumulation Studies

**Figure 5:**
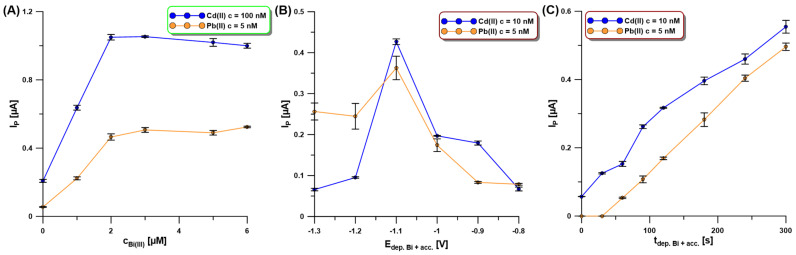
The influence of Bi(III) concentration (**A**), E_dep.Bi+acc._ (**B**), and t_depBi.+acc._ (**C**) on the SWASV response of Cd(II) (100 nM—(**A**), and 10 nM—(**B**,**C**)) and Pb(II) (5 nM—(**A**–**C**)). The SWASV conditions: 0.2 M acetate buffer of pH = 4.0, f of 25 Hz, E_SW_ of 25 mV, and ΔE of 4 mV.

Our SEM studies confirmed that the activation of the electrode in 0.1 M PBS of pH = 7 contributes to a change in the morphology of the deposited bismuth film. Moreover, the Bi(III) concentration as well as the potential and time of simultaneous Bi deposition and Cd and Pb accumulation (E_dep.Bi+acc._ and t_dep.Bi+acc._) influence the morphology of the bismuth film and consequently the Cd and Pb signals. The Bi(III) content in 0.2 M acetate buffer of pH = 4.0 was studied for the Cd(II) and Pb(II) concentrations of 100 nM and 5 nM, respectively (Figure 5A). The current intensity of both cadmium and lead increased with the increase in Bi(III) concentration up to 3.0 µM um and then the signals were practically constant. This is most probably related to the fact that initially the active surface of the electrode increases, i.e., the number of active sites on which Cd and Pb can accumulate increases, but then the active surface is practically constant.

In the next stage of the research, the influence of the potential and time of simultaneous Bi deposition and Cd and Pb accumulation (E_dep.Bi+acc._ and t_dep.Bi+acc._) on the SWASV response of Cd(II) (10 nM) and Pb(II) (5 nM) was examined. The E_dep.Bi+acc._ was changed from −1.3 to −0.8 V for 120 s of t_dep.Bi+acc._ (Figure 5B). In the presented figure, it can be seen that the highest signals of both metals were obtained using the accumulation potential of −1.1 V. Then, the t_dep.Bi+acc._ was changed in the range from 0 to 300 s (Figure 5C). It can be stated that without using bismuth modification, only a slight Cd signal is visible, with no lead signal. Analytical signals of Cd and Pb increase practically linearly with an increasing time of the simultaneous deposition of the bismuth film and accumulation of the analytes. However, due to the shortening of the analysis time itself for further measurements, the time of 120 s was chosen.

### 2.4. Effect of Technique Parameters

**Figure 6:**
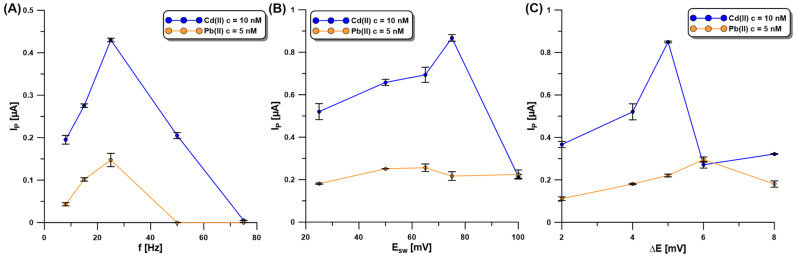
The influence of f (**A**), E_SW_ (**B**), and ΔE (**C**) on the SWASV response of Cd(II) (10 nM) and Pb(II) (5 nM) at the aGCE/BiF. The SWASV conditions: 0.2 M acetate buffer of pH = 4.0, 3.0 µM Bi(III), electrochemical cleaning of the electrode surface at 0.5 V for 60 s, E_dep.Bi+acc._ of −1.1 V, and t_dep.Bi+acc._ of 120 s.

For achieving optimum sensitivity, the SWASV technique was selected and its parameters (the frequency—f, the amplitude—E_SW_, and the step potential—ΔE) optimized. f was investigated in the range of 8–75 Hz at a constant E_SW_ of 25 mV and ΔE of 4 mV (Figure 6A). The Cd (10 nM) and Pb (5 nM) signals reached extreme values for the f of 25 Hz, so this value was approved for further investigation. In the subsequent step of the experiments, the influence of E_SW_ (25–100 mV) on the SWASV responses of Cd(II) (10 nM) and Pb(II) (5 nM) at the aGCE/BiF was studied (Figure 6B). With the increase in E_SW_, the SWASV responses of Cd(II) increased, reaching the highest intensity at the E_SW_ of 75 mV. On the other hand, the Pb(II) signal reached its maximum value at the E_SW_ equal to 50 mV. As a compromise between both responses, the E_SW_ value of 50 mV was selected. Then, changes in ΔE (2–8 mV), with an f of 25 Hz and E_SW_ of 50 mV as the selected values, were made to study their influence on the SWASV responses of Cd(II) (10 nM) and Pb(II) (5 nM) (Figure 6C). The Cd peak current increased with an increasing ΔE up to 5 mV, while the Pb peak current increased to a ΔE value of 6 mV. As a compromise between the obtained results, the ΔE equal to 5 mV was chosen for further studies.

### 2.5. Electrochemical Cleaning, Calibration, Sensitivity, and Application

Repeatability was evaluated with ten successive SWASV measurements of 10 nM Cd(II) and 5 nM Pb(II) at one aGCE/BiF. The different procedures of electrode surface cleaning between each measurement (0.5 V for 60 s, 1.0 V for 20 s, and 30 times −1.1 V for 1 s and 0.5 V for 1 s) were evaluated. A significant improvement in signal reproducibility was observed for n = 10, when changing potentials were repeated 30 times: −1.1 V for 1 s and 0.5 V for 1 s (RSD for Cd(II): 2.8 vs. 5.7 vs. 1.1%, RSD for Pb(II): 23.8 vs. 10.1 vs. 3.1%). Reproducibility was calculated based on the signals (n = 9) of 10 nM Cd(II) and 5 nM Pb(II) registered at three independently prepared aGCE/BiFs, and RSD values of 5.2% for Cd(II) and 6.3% for the Pb(II) were obtained.

**Figure 7:**
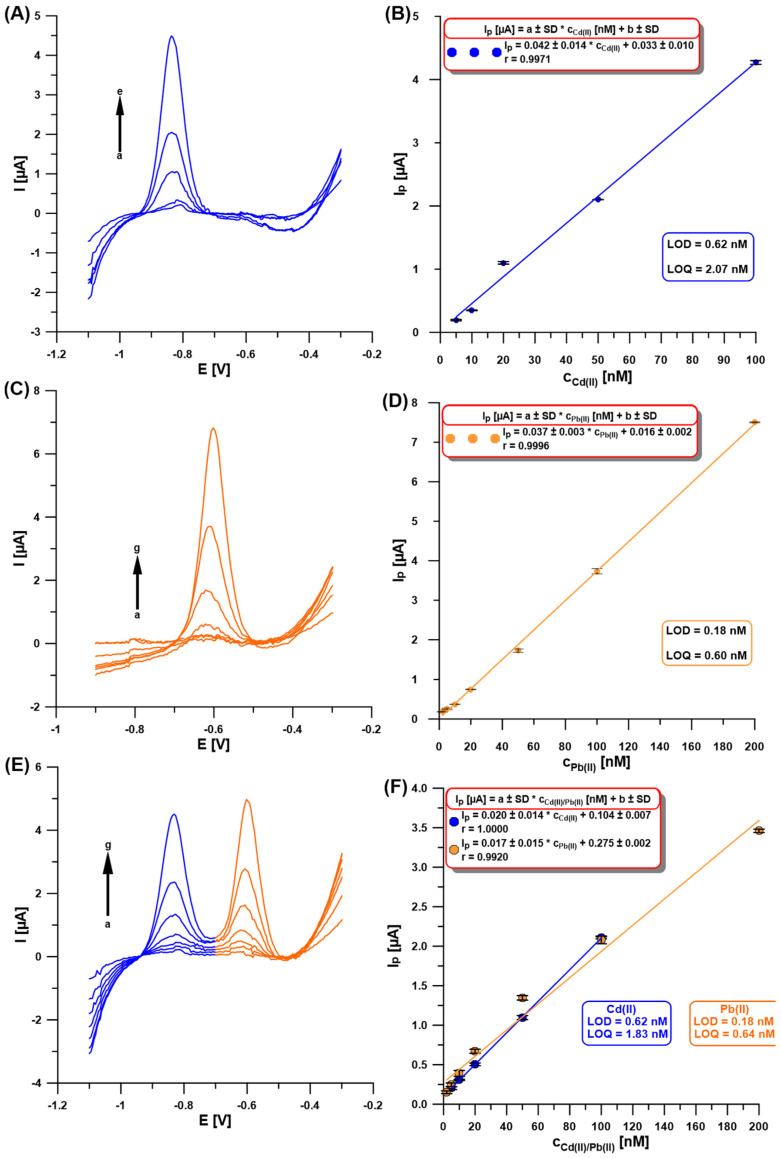
The SWASVs registered at the aGCE/BiF in 0.2 M acetate buffer of pH = 4.0 containing 0.3 µM Bi(III) and (**A**) Cd(II) (a → e, 5–100 nM), (**C**) Pb(II) (a → g, 2–200 nM), (**E**) Cd(II) and Pb(II) (a → g, 5–100 nM, and 2–200 nM, respectively). The calibration plots of (**B**) Cd(II), (**D**) Pb(II), and (**F**) Cd(II) and Pb(II). The SWASV conditions: the electrochemical cleaning step—changing potentials repeated 30 times (−1.1 V for 1 s and 0.5 V for 1 s), E_dep.Bi+acc._ of −1.1 V, t_dep.Bi+acc._ of 120 s, f of 25 Hz, E_SW_ of 50 mV, and ΔE of 5 mV. The baseline was subtracted from each measurement.

Once the experimental conditions were optimized, the SWASVs for individual Cd(II) and Pb(II), and simultaneous determination of Cd(II) and Pb(II), were recorded (Figure 7A,C,E). As shown in Figure 7B,D,F, the obtained linearity of the calibration curves was very good and extended up to 100 nM for Cd(II) and 200 nM for Pb(II), with the low limits of detection (LOD) and quantification (LOQ) of 0.62 and 0.18 nM, and 2.07 and 0.60 nM, respectively [30]. Table 1 compares the developed procedure for the determination of Cd(II) and Pb(II) using the aGCE/BiF with others described in the literature [9,10,11,12,13,14,15,19,20,21,22,23,24,25,26,27,28]. Without any doubt, it can be stated that the developed procedure offers wide linear ranges of calibration curves and one of the lowest detection and quantification limits for Cd(II) and Pb(II) compared to those described in the literature. In addition, the obtained sensor is environmentally friendly and does not require the use of a wide range of reagents or complicated preparation work.

The selectivity of the proposed SWASV procedure at the aGCE/BiF was examined by studying the 5 nM Cd(II) and 5 nM Pb(II) responses without and in the presence of metal ions (Fe(III), V(V), Cu(II), Zn(II), Mg(II), Mo(VI), Ni(II), Se(VI), Tl(I), and Co(II)) and Triton X-100 (0.2–2.0 ppm); Triton X-100 added to the supporting electrolyte has a similar influence on the voltammetric response as surfactants in natural waters. It was found that the addition of Triton-X caused a rapid increase in the Cd and Pb signals to over 200% of their original values, which confirms the necessity of pretreatment of water samples, e.g., due to the need for mineralization of the water samples (decomposition of the organic matrix). Moreover, it was found that a 10-fold excess of the tested metal ions does not cause a change in the analytical signals of Cd and Pb of greater than 15% compared to the original values.

To evaluate the capabilities of the proposed methodology for Cd(II) and Pb(II) determination in water samples, the certified reference material (TMRAIN-04, rainwater) was analyzed in triplicate. The obtained results were compared to the certified values, and statistical analysis was performed. Finally, it was found that Δm ≤ U_Δ_, so there is no significant difference between the measured results and the certified value at a 95% confidence level (coverage factor, k = 2) (Table 2) [31], and therefore the proposed SWASV procedure at the aGCE/BiF can be applied for Cd(II) and Pb(II) analysis of water samples.

Moreover, the developed procedure was applied to the analysis of river water samples, and the determined values of Cd(II) and Pb(II) were 9.50 ± 0.48 nM and 12.27 ± 0.65 nM, respectively. The values of recoveries (samples enriched with 10 nM Cd(II) and Pb(II)) were 107% of Cd(II) and 109% for Pb(II), respectively.

## 3. Materials and Methods

### 3.1. Instrumentation

For the electrochemical experiments, a potentiostat/galvanostat (µAutolab, Utrecht, The Netherlands, Eco Chemie) coupled with GPES 4.9 software (voltammetric studies) was used. Commercially available glassy carbon electrodes (geometric area of 3.14 mm^2^, Mineral, Warsaw, Poland) were polished using 0.3 µm alumina slurry on a Buehler polishing pad (Lake Bluff, IL, USA). The measurements were performed in a three-electrode configuration consisting of the activated glassy carbon electrode with an electrochemically deposited bismuth film (aGCE/BiF, working), a silver chloride electrode (3 M KCl, reference), and a Pt wire (auxiliary electrode). Microscopic images of the GCE/BiF and aGCE/BiF surfaces were obtained using a scanning electron microscope (SEM, high-resolution, Quanta 3D FEG, FEI, Hillsboro, OR, USA). The certified reference materials were mineralized using a UV digester purchased from Mineral, Warsaw, Poland.

### 3.2. Reagents

The stock standard solutions of Pb(II), Cd(II), and Bi(III) (1 g/L, Merck, Darmstadt, Germany) were dissolved in 0.1 M HNO3 (Merck, Darmstadt, Germany) to the required dilution. A 0.1 M PBS (phosphate-buffered saline) solution of pH = 7 was used for the electrochemical activation of the GCE surface. Non-aerated 0.2 M acetate buffer (pH = 4.0 ± 0.1) was used as a supporting electrolyte (prepared from CH3COOH and NaOH, Merck Merck, Darmstadt, Germany). We prepared 1 mM solutions of Fe(III), V(V), Cu(II), Zn(II), Mg(II), Mo(VI), Ni(II), Se(VI), Tl(I), and Co(II) from reagents bought from Merck in ultra-purified water (>18 MΩ cm, Milli-Q system, Millipore, Gillingham, UK). Moreover, to study the interference effect, a Triton X-100 (Fluka, Gillingham, UK) was used. Certified reference material (CRM), rainwater TMRAIN-04, was obtained from Environment Canada (Burlington, ON, Canada). The CRM and river water (Poland) samples were mineralized by UV irradiation for 3 h. Then, 6.5 mL of the sample was introduced into the electrochemical cell, and the voltammograms were registered.

### 3.3. GCE Surface Activation and SWASV Measurements

Before the series of SWASV measurements, the GCE surface was pre-anodized in a 0.1 M PBS solution of pH = 7 using five cyclic voltammetric (CV) scans in the potential range of −1.5–2.5 V at a scan rate (ν) of 100 mV/s [18]. Then, the Cd(II) and Pb(II) measurements were performed in 0.2 M acetate buffer (pH = 4) containing 3.0 µM Bi(III). The optimized SWASV parameters were as follows: the electrochemical cleaning step—30 times (−1.1 V) for 1 s and 0.5 V for 1s; simultaneous Bi deposition and Cd and Pb accumulation at −1.1 V (E_dep.Bi+acc_.) for 120 s (t_dep.Bi+acc_.); the frequency (f) of 25 Hz; the amplitude (ESW) of 50 mV; and the step potential (ΔE) of 5 mV. The baseline was subtracted from each measurement. The average peak current values are shown with standard deviation (SD) for three measurements.

## 4. Conclusions

The investigation performed in this study shows the promising role of the activated glassy carbon electrode with an in situ deposited bismuth film (aGCE/BiF) to individually and simultaneously determine the nanotrace levels of Cd(II) and Pb(II) by square-wave anodic stripping voltammetry (SWASV). The results demonstrated that the simple procedure of activation (0.1 M phosphate-buffered saline (PBS) of pH = 7, five cyclic voltammetric scans in the range of −1.5–2.5 V at ν of 100 mV/s) before the series of measurements contributes to changes in the morphology of the deposited bismuth film, increasing the active surface of the electrode and improving the rate of charge transfer between the electrode and the solution. The developed procedure offers wide linear ranges of calibration curves (5–100 nM for Cd(II), and 2–200 nM for Pb(II)) and some of the lowest detection and quantification limits for Cd(II) and Pb(II) (0.62 and 0.18 nM, and 2.07 and 0.60 nM, respectively) compared to those described in the literature. In addition, the obtained sensor is environmentally friendly and does not require the use of a wide range of reagents or complicated preparation work. The newly prepared method was used to determine the amounts of Pb(II) and Cd(II) in the certified reference material, and the results were in agreement with the certified values. Moreover, the procedure was successfully applied to determine Cd(II) and Pb(II) in river samples. The satisfactory accuracy and precision of the SWASV procedure were confirmed in our statistical evaluation.

## Figures and Tables

**Table 2:** CRM (TMRAIN-04) analysis.

Analytical Parameter	Cd(II)	Pb(II)
Measured value ± SD ^a^ (µg/L)	0.508 ± 0.0254	0.312 ± 0.0172
Certified value ± 2σ ^b^ (µg/L)	0.524 ± 0.0602	0.346 ± 0.0695
Δm ^c^	0.016	0.034
UΔ ^d^	0.062	0.121

^a^ SD for n = 3. ^b^ 2-sigma limit for an individual measurement. ^c^ absolute difference between the mean measured value and the certified value. ^d^ expanded uncertainty of the difference between the results and the certified value.

## Data Availability

The original contributions presented in this study are included in the article. Further inquiries can be directed to the corresponding author.
